# The Interplay between von Hippel–Lindau Tumor Suppressor Gene, Lon Protease, ROS Accumulation, and Inflammation in Clear Cell Renal Cell Carcinoma

**DOI:** 10.3390/cimb46100671

**Published:** 2024-10-11

**Authors:** Yao-Chou Tsai, Chan-Yen Kuo

**Affiliations:** 1 Division of Urology, Department of Surgery, Taipei Tzuchi Hospital, The Buddhist Tzu Chi Medical Foundation, New Taipei City 23142, Taiwan; tsai1970523@yahoo.com.tw; 2 School of Medicine, Buddhist Tzu Chi University, Hualien 970374, Taiwan; 3 Department of Research, Taipei Tzu Chi Hospital, Buddhist Tzu Chi Medical Foundation, New Taipei City 23142, Taiwan; 4 Department of Nursing, Cardinal Tien College of Healthcare and Management, New Taipei City 23142, Taiwan

**Keywords:** von Hippel–Lindau, clear cell renal carcinoma, reactive oxygen species, Lon protease, inflammation

## Abstract

This study explores the role of the von Hippel–Lindau (VHL) tumor suppressor gene and Lon protease in the development of clear cell renal carcinoma (ccRCC) through mechanisms involving inflammation and reactive oxygen species (ROS) accumulation in kidney cells. By examining the impact of VHL on the early stages of kidney cancer development, this research highlights the contributions of inflammation and ROS, as well as the involvement of Lon protease. The findings reveal increased Lon expression and ROS levels in VHL-knockdown HK-2 cells, along with elevated phospho-c-Jun N-terminal kinase (JNK) levels, emphasizing the complex interplay between VHL, Lon protease, inflammation, and ROS in kidney cell models. These insights point to potential therapeutic pathways for ccRCC.

## 1. Introduction

Clear cell renal carcinoma (ccRCC) is the most common subtype of renal cell carcinoma, typically characterized by the loss of function of the tumor suppressor gene von Hippel–Lindau (VHL) in the majority of cases [1]. The VHL protein is crucial in regulating the stability of hypoxia-inducible factors (HIFs), which are key transcription factors involved in cellular responses to hypoxia [2]. Mutations in VHL lead to the dysregulation of HIFs, resulting in the abnormal expression of genes involved in angiogenesis, glycolysis, and cell proliferation, thereby promoting tumorigenesis [3]. However, the precise mechanisms by which VHL loss promotes the early stages of kidney cancer development remain unclear.

Previously, we explored the impact of VHL gene inactivation on early-stage kidney cancer, focusing on inflammation through the activation of the IRE1α signaling pathway associated with endoplasmic reticulum stress [4]. We also investigated the role of VHL in ccRCC development via the modulation of reactive oxygen species (ROS) and lipocalin 2 (LCN2)-dependent inflammatory responses in renal tubular cells [5], as well as the polarization of macrophage RAW 264.7 cells [6]. The findings of the above work underscore the importance of understanding the molecular mechanisms underlying inflammation and ROS accumulation in VHL-deficient renal tubular cells for the development of targeted therapies for kidney diseases.

Lon protease (Lon) preferentially degrades oxidized mitochondrial aconitase through an ATP-stimulated mechanism [7]. Recent studies have illuminated its multifaceted roles in various cellular processes, including protein turnover, the stress response, and apoptosis regulation, suggesting that it contributes to cancer progression and therapeutic resistance [8,9]. Under acute stress, Lon is highly inducible and confers a protective effect, while, under chronic stress conditions, the Lon levels decline [10]. The dysregulation of Lon protease has been implicated in various mitochondrial disorders and age-related diseases, highlighting its significance in mitochondrial quality control [11,12]. Our previous research indicated that increased Lon protease expression promotes the survival and aggressive behavior of cancer cells by facilitating ROS generation through mitochondrial complex I activity in various cancer types [13]. Lon plays a key role in regulating cancers such as colon cancer, melanoma, and cervical cancer [13,14,15]; however, its role in ccRCC remains largely unknown.

In the work described in this brief report, we utilized HK-2 cells, derived from human renal proximal tubule cells (representing “normal” renal proximal tubule cells) [16], with shRNA-mediated VHL inactivation as a model system to investigate Lon’s involvement in regulating ROS accumulation. Additionally, the c-Jun N-terminal kinases (JNKs) play a key role in the regulation of tumorigenesis via activated chronic inflammation [17]. We also evaluated the effect of a VHL mutant on phosphor-JNK expression.

## 2. Materials and Methods

### 2.1. Antibodies

The antibodies used for Western blot analysis included rabbit polyclonal antibodies against VHL (#A0377, 1:1000 dilution; ABclonal, Woburn, MA, USA), Lon (#A4293, 1:1000 dilution; ABclonal, Woburn, MA, USA), phosho-JNK (#AP1337, 1:1000 dilution; ABclonal, Woburn, MA, USA), JNK (#A4867) (1:1000 dilution; ABclonal, Woburn, MA, USA), and β-actin (#AC038, 1:5000 dilution; ABclonal, Woburn, MA, USA).

### 2.2. Cell Culture

Human renal proximal tubular epithelial (HK-2) and human embryonic kidney cells 293 (HEK 293) cells were obtained from the Bioresource Collection and Research Center (BCRC, Hsinchu, Taiwan) and Millipore (Billerica, MA, USA), respectively. The cells were cultured in T-75 flasks (Corning, Waltham, NY, USA) using DMEM/Ham’s F12 medium (Gibco, NY, USA) for HK-2 and DMEM for HEK 293 (Gibco, New York, NY, USA) supplemented with 10% fetal bovine serum, 25 mM D-glucose, 2 mM L-glutamine, 1 mM sodium pyruvate, and penicillin–streptomycin (50 U/mL; Sigma, St. Louis, MO, USA).

### 2.3. Transfection

HK-2 or HEK 293 cells were transfected with a vector control (scramble) or a vector expressing shRNAs targeting VHL (shVHL), generously provided by Dr. T. Hsu (Department of Biomedical Sciences and Engineering, National Central University, Jhongli, Taiwan). Transfection was performed using the BTX™ Gemini X2 Electroporation System (BTX, Holliston, MA, USA), following the manufacturer’s protocol. Briefly, 5 × 10^5^ cells were transfected with 4 μg of plasmid DNA using a single 100 V pulse for 10 milliseconds and then plated in 6-well plates. To assess the transfection efficiency, the electroporated cells were cultured for 48 h, followed by Western blot analysis to detect VHL expression.

### 2.4. Detection of ROS Levels

Intracellular ROS production was measured using the 2′,7′-dichlorofluorescin diacetate reagent (Sigma-Aldrich, St. Louis, MO, USA). Following trypsinization, the cells were collected and the ROS levels were assessed via flow cytometry (FACScan, Becton Dickinson, Franklin Lakes, NJ, USA) with an excitation wavelength of 498 nm and an emission wavelength of 522 nm.

### 2.5. Protein Extraction and Western Blot Analysis

Protein extraction was carried out using a radioimmunoprecipitation assay (RIPA) lysis buffer supplemented with protease and phosphatase inhibitor cocktails (Roche, Basel, Switzerland), with the cells incubated on ice for 30–60 min. The protein concentration in each sample was quantified using a BCA assay kit (Thermo Fisher Scientific, Waltham, MA, USA).

For the Western blot analysis, proteins were separated using SDS-polyacrylamide gel electrophoresis and subsequently transferred onto PVDF membranes with a 0.45 µm pore size. The membranes were then blocked with 5% milk or bovine serum albumin (Sigma) in 1xPBST buffer for 30–60 min. Primary antibodies, including anti-β actin (Cell Signaling, Danvers, MA, USA), were incubated with the membranes overnight at 4 °C. The membranes were washed three times for 10 min each with 1xPBST buffer on alternate days, followed by incubation with a horseradish peroxidase (HRP)-conjugated secondary antibody for 60 min at room temperature. Afterward, the membranes were washed again three times for 10 min each with 1xPBST buffer.

The protein levels were detected using the Cytiva Amersham™ ECL™ Prime Western Blotting Detection Reagent (Thermo Fisher Scientific, Waltham, MA, USA) and visualized with a ChemiDoc™ XRS + System (Bio-Rad Laboratories, Hercules, CA, USA). The intensities of the protein bands were quantified using the ImageJ (V 1.8.0) software (National Institutes of Health, Baltimore, MD, USA).

### 2.6. Statistical Analysis

Statistical analyses were performed using the GraphPad Prism 5.0 for Windows (Version 5.04) software (Boston, MA, USA), with a *p*-value of <0.05 considered statistically significant. Multiple group comparisons were conducted using a one-way ANOVA followed by Tukey’s post hoc test. Data are presented as the mean ± standard error of the mean (SEM), and statistical significance was defined as a *p*-value of <0.05 for all tests.

## 3. Results

**Figure 1:**
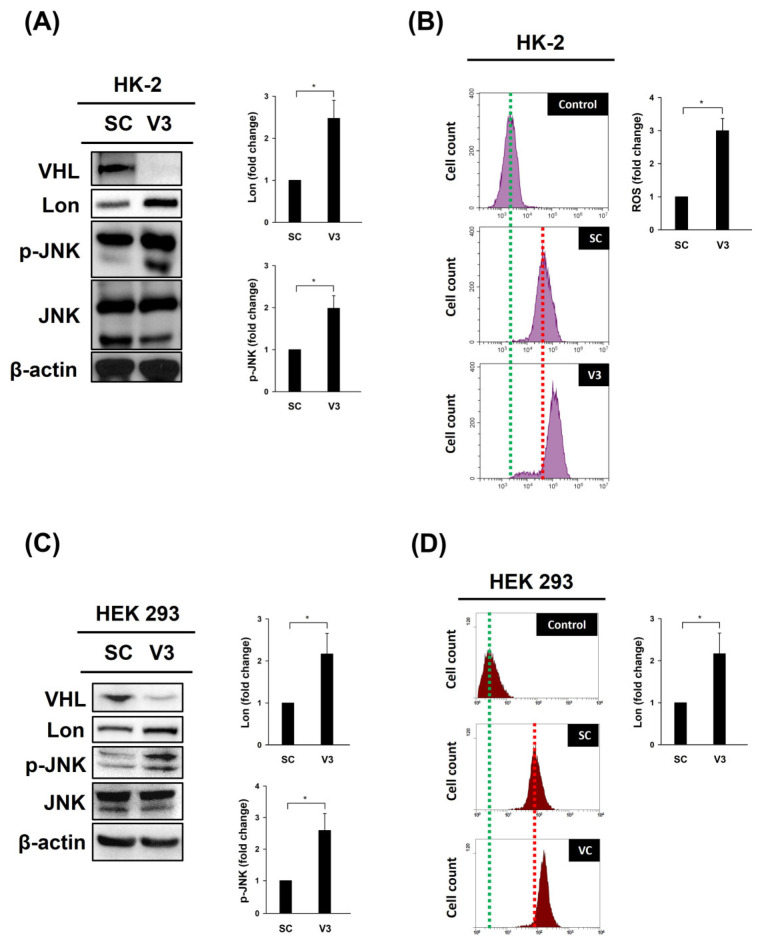
*VHL* knockdown mediates the expression of Lon and phospho-JNK (p-JNK) and ROS accumulation. HK-2 or HEK-293 cells were transfected with a vector expressing a scrambled shRNA (SC) or an shRNA specific to VHL, shVHL3 (V3), as described in a previous study [4], by electroporation (Gemini X2 Electroporation System; BTX, Holliston, MA, USA) and incubated for 48 h. (**A**,**C**) Western blot analysis of the cell lysate using the indicated antibodies. β-Actin was used as a loading control. VHL was successfully knocked down (top). (**B**,**D**) 2′,7′-Dichlorodihydrofluorescein (10 μM DCF-DA, Santa Cruz, Dallas, TX, USA) was used to determine the intracellular ROS levels after transfection. A flow cytometry analysis (BD Biosciences, San Jose, CA, USA) was used to detect the fluorescence intensity. Quantitative images demonstrating the levels of specific proteins or ROS determined using ImageJ software. Error bars display standard deviations from three independent replications (n = 3). * *p* < 0.05.

Lon was upregulated and the ROS levels increased in VHL-knockdown HK-2 (Figure 1A,B) and HEK 293 (Figure 1C,D) cells. Additionally, phosphor-JNK, a marker of inflammation [18], was upregulated in VHL-knockdown HK-2 (Figure 1A) and HEK 293 (Figure 1C) cells. These findings suggest that VHL plays a critical role in regulating inflammation and ROS accumulation in HK-2 (Figure 1B) and HEK 293 (Figure 1D) cells. 

## 4. Discussion

ROS play a vital role in the progression of chronic inflammation and tumor metastasis [19]. This short report describes the role of the von Hippel–Lindau (VHL) tumor suppressor gene and Lon protease in clear cell renal carcinoma (ccRCC) development, focusing on inflammation and reactive oxygen species (ROS) accumulation in kidney cells. The research highlights the contributions of VHL loss to increased inflammation and ROS, alongside elevated Lon protease and phospho-JNK levels, in VHL-knockdown HK-2 cells. These findings suggest a complex interplay between VHL, Lon protease, inflammation, and ROS, offering potential therapeutic pathways for ccRCC.

A previous article described a study on clear cell renal cell carcinoma (ccRCC), focusing on how the loss of the von Hippel–Lindau (VHL) gene in cancer cells impacts the tumor microenvironment (TME), particularly immune cell infiltration and behavior. Moreover, the results indicated that the tumor-associated macrophages (TAMs) in these tumors showed increased glucose consumption, phagocytosis, and inflammatory activity, while lymphocytes exhibited reduced activation and a weaker response to anti-PD-1 therapy. The chemokine CX3CL1, linked to VHL deficiency, was identified as a key factor in promoting myeloid cell infiltration [20]. Therefore, a VHL-deficient mutant may reprogram the tumor immune landscape, offering insights into potential therapeutic strategies for ccRCC. Moreover, Nguyen-Tran et al. reported that Oncostatin M (OSM), secreted by VHL-deficient renal cells, activates endothelial cells (ECs), which then promote inflammation and tumorigenesis in the development of ccRCC [21]. On the other hand, Nguyen et al. found that VHL-deficient kidney cells secrete IL-6, which recruits macrophages and promotes their polarization towards a protumorigenic M2 phenotype. These activated macrophages, in turn, secrete CCL-18 and TGF-β1, which drive epithelial-to-mesenchymal transition (EMT) and tumor progression. These findings highlight the importance of OSM, IL-6, and CCL-18 in the crosstalk between tumor cells and the immune microenvironment, offering potential therapeutic targets for early intervention in ccRCC [22]. 

Mitochondrial Lon, a chaperone protein, promotes cancer metastasis by increasing mitochondrial reactive oxygen species (ROS) production [11,23]. A recent study shows that elevated Lon triggers epithelial-to-mesenchymal transition (EMT) through the ROS-dependent activation of the p38 and NF-κB signaling pathways. Lon also interacts with pyrroline-5-carboxylate reductase 1 (PYCR1), leading to increases in ROS, EMT, and the production of inflammatory cytokines to promote angiogenesis, EMT, and M2 macrophage polarization. Additionally, Lon expression enhances the immunosuppressive tumor microenvironment by further promoting M2 macrophage activation. These findings suggest that targeting the mitochondrial redox balance could offer a potential therapeutic approach to enhance T cell function in cancer immunotherapy. 

Reactive oxygen species (ROS) act as a double-edged sword in carcinogenesis, both promoting and inhibiting malignant behavior and cancer progression via reducing oxidative stress and increased ROS accumulation, respectively [24]. The effects of ROS, whether produced by tumor cells or cells within the tumor microenvironment (TME), vary widely depending on their levels, locations, and regulatory mechanisms [25]. Previous research showed that VHL mutations lead to excessive reactive oxygen species (ROS) levels and chronic inflammation, contributing to ccRCC [4]. Using cell models with VHL mutations, the study explored how VHL influenced ROS accumulation and the role of lipocalin 2 (LCN2) in regulating inflammation and macrophage recruitment. The findings revealed that VHL mutations increased ROS production, which could be mitigated by LCN2 knockdown. VHL also regulates LCN2 expression and secretion, suggesting that LCN2 plays a key role in VHL-mediated ROS production and inflammation [5]. Furthermore, the results showed that VHL mutations in kidney cells increase reactive oxygen species (ROS) and decrease the expression of the antioxidant enzyme GPX4, promoting ferroptosis in an LCN-2-dependent manner. Additionally, LCN-2 was shown to heighten inflammation and promote M1-like polarization in macrophages [6]. However, further studies are warranted to explore the relationship between VHL and Lon in the development of ccRCC, which could provide valuable insights into potential therapeutic targets for this malignancy.

Principal Component Analysis (PCA), as a dimensionality reduction technique, would indeed help to simplify the complexity of our high-dimensional gene expression data and allow us to capture the major sources of variance between samples [26]. This could offer a clearer distinction between different experimental conditions or sample groups, and we will consider integrating PCA to enhance our analysis [27]. Similarly, Support Vector Machine (SVM), being a robust supervised classification tool, could be highly beneficial for classifying samples into distinct categories (e.g., cancerous vs. non-cancerous). Its application could refine our predictive modeling and help identify key genes that contribute to classification decisions [28,29]. Therefore, in future iterations of this study or subsequent publications, we plan to integrate these approaches to further validate and enrich our findings.

## Data Availability

The authors declare that all data supporting the findings of this study are available within the paper and any raw data can be obtained from the corresponding author upon request.
